# Excellent Recovery of Shoulder Movements After Decompression and Neurolysis of Long Thoracic Nerve in Teen Patients With Winging Scapula

**Published:** 2019-04-25

**Authors:** Rahul K. Nath, Chandra Somasundaram

**Affiliations:** Texas Nerve and Paralysis Institute, Houston

**Keywords:** long thoracic nerve injury, winging scapula, decompression and neurolysis, shoulder movements, active range of motion

## Abstract

**Introduction:** In teens, athletes, in general, have been found to have shoulder pain and or winging scapula resulting from long thoracic or spinal accessory nerve injuries. Accident (fall) and stretch injuries due to overuse and poor sports techniques mainly cause these injuries that affect their upper extremity movements and functions. Here, we report a significant improvement in scapula winging and shoulder active range of motion in 16 teen patients after long thoracic nerve decompression and neurolysis. **Patients and Methods:** This was a retrospective study of 16 teen patients who had severe winging scapula and poor shoulder movements and function. Therefore, they underwent decompression and neurolysis of long thoracic nerve with us, between 2005 and 2016. The average patient age was 17 years (range, 14-19 years). These patients had been suffering from paralysis for an average of 15 months (range, 2-48 months). All patients underwent a preoperative electromyographic assessment in addition to clinical evaluation to confirm the long thoracic nerve injury. **Results:** Scapula winging was severe in 10 of 16 patients (63%), moderate in 2 patients (12%), and mild in 4 patients (25%) in our present study. Mean shoulder abduction (128°) and flexion (138°) were poor preoperatively. Shoulder abduction and flexion improved to 180° in 15 patients (94%) and good (120°) in 1 patient (6%) at least 2 months after surgery. In 11 patients (69%), the winged scapula was completely corrected postsurgically and it was less prominent in other 5 patients. **Conclusion:** Long thoracic nerve decompression and neurolysis significantly improved scapular winging in all 16 teen patients in our present study, producing “excellent” shoulder movements in 15 patients (94%) and “good” result in 1 patient (6%).

Long thoracic nerve (LTN) injury is a rare and misdiagnosed painful condition that causes scapular instability and loss of upper extremity movements and function in some patients. Scapular winging results from nerve damage to the LTN, spinal accessory nerve, or, less commonly, the dorsal scapular nerve. C5-C7 brachial plexus preganglionic injuries are also associated with complete paralysis of the LTN.^1^ Several athletic activities are known to cause injury to LTN.^2^

Most of our teen patients in this study had sports-related injuries, because of overuse and practicing poor sports technique,^3^ as well as accidents. Moreover, they are skeletally immature.^3^ As the LTN passes along the thorax, it can be compressed by blood vessels and/or fibrotic tissue.^4^ Although some investigators have recommended conservative treatment of this condition,^5^ often timely surgical treatment^6^ is vital to the recovery of scapular winging secondary to LTN palsy.^7^ Recently, we have analyzed and compared the effectiveness of muscle and tendon operative procedures to decompression and neurolysis of LTN and demonstrated that LTN decompression is an easy and effective treatment to correct winging scapula.^8^ Here, we report a significant improvement in scapula winging and active range of motion (AROM) of the shoulder in 16 teen patients after LTN decompression and neurolysis.

## PATIENTS AND METHODS

Of 555 patients who visited our clinic with winging scapula since 2005, 16 teenage patients met the inclusion criteria of this study.

### Inclusion criteria

1. Teen patients (aged between 13 and 19 years) who had winging scapula were clinically evaluated by the lead author (R.K.N.) and LTN injury was confirmed by electromyography (EMG).
2. Teen patients who had LTN decompression and neurolysis procedure at our clinic.

### Exclusion criteria

1. Teen patients who had LTN injury and winging scapula and who were included in our previously published studies.^9^^-^11
2. Teen patients who have no postoperative follow-up.

**Figure 1:**
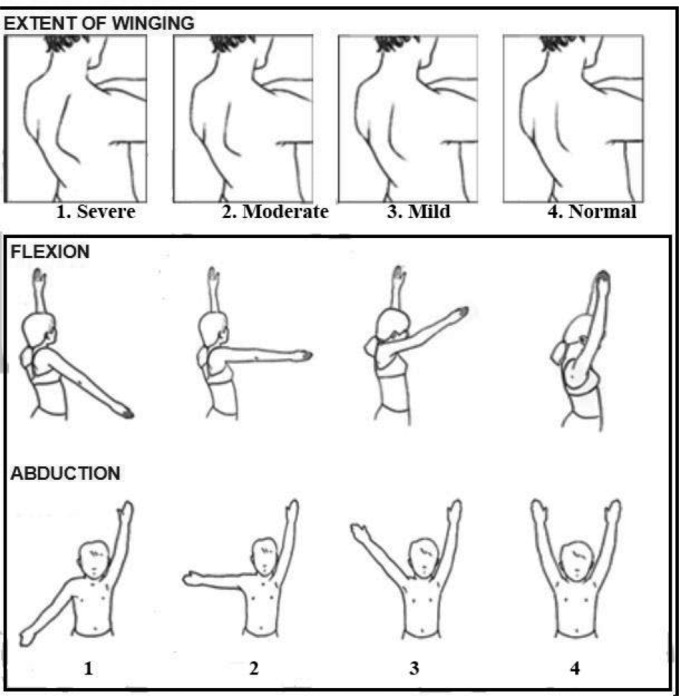
Extent of scapular winging range, 1-4: 1, severe; 2, moderate; 3, mild; 4, normal.

This was a retrospective study of 16 teen patients who had winging scapula and therefore underwent LTN decompression between 2005 and 2016, as described in our previous publications.^9^^,^^10^ The average patient age was 17 years (range, 14-19 years). Patients had been suffering from paralysis for an average of 1.3 years (range, 2-48 months). All patients underwent a preoperative electromyographic assessment in addition to clinical evaluation to confirm the LTN injury. We adopted a 4-point numerical scale for determining the extent of scapula winging: 1, severe; 2, moderate; 3, mild; and 4, normal as shown in Figure 1. The angle of humeral elevation before and after surgical neurolysis of the LTN was captured on the video. Stills were taken from the video of the patients performing abduction, and the angle between a line parallel to the medial line and the humerus was measured (0° being relaxed at the side and 180° being fully abducted above the head). The paired Student *t*-test statistics was applied to compare the pre- and postoperative mean Mallet and supination scores using the Analyze it plugin (Leeds, UK) for Microsoft Excel 2003. A value of *P* < .05 was considered statistically significant.

This was a retrospective study of patient charts, which exempted it from the need for institutional review board approval in the United States. Patients were treated ethically in compliance with the Helsinki declaration. Documented informed consent was obtained for all patients.

## RESULTS

Of 16 teen patients in our present study, 9 patients (56%) had a sports-related injury and 5 patients (31%) had accidents (3 motor vehicle accidents and 2 falls). In the other 2 patients (13%), the cause of the injury was not known (Table 1). Scapula winging was severe in 10 of 16 patients (63%), moderate in 2 patients (12%), and mild in 4 patients (25%) in our present study. Mean shoulder abduction and flexion were preoperatively 128° and 138°, respectively (Table 2).

**Figure 2:**
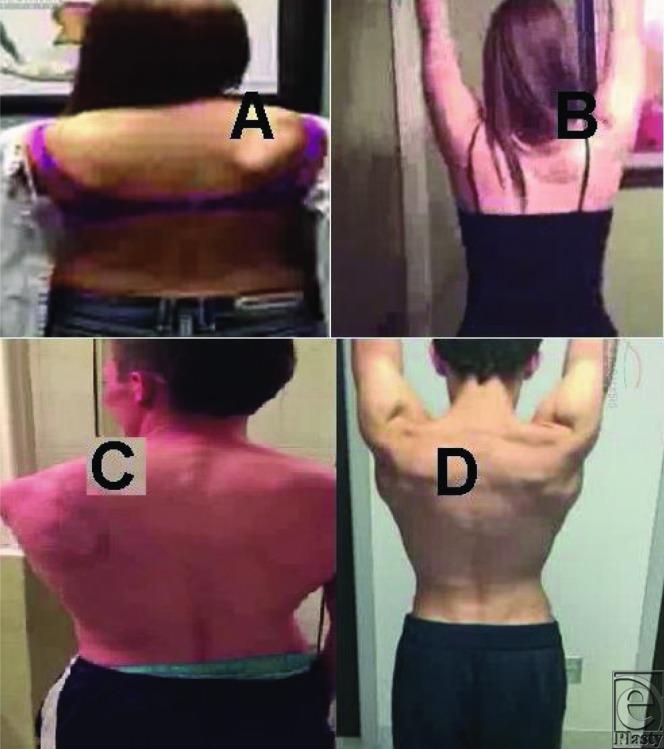
Excellent recovery of shoulder movements (abduction 180°) after LTN decompression and neurolysis. (A, B) Photographs of a female teen patient (16.5 years old) who had winging scapula due to an accident or fall. (A) Preoperative photograph showing clearly winged scapula. (B) Post-LTN decompression photograph showing excellent recovery and full AROM (shoulder abduction 180°). (C, D) Photographs of a male teen patient (19 years old) who had winging scapula due to a motor vehicle accident. (C) Preoperative photograph showing winging scapula. The patient recovered full AROM (shoulder abduction 180°) 2 months after LTN decompression (D). LTN indicates long thoracic nerve; AROM, active range of motion.

All patients showed some improvements in shoulder movements on the next day of the LTN decompression procedure. The improvement in shoulder AROM was excellent (180°) in 15 patients (94%) and good (120°) in 1 patient (6%) after at least 2 months of post-LTN decompression follow-up (Fig 2). The winged scapula was completely corrected in 11 patients (69%), and it was less prominent in other 5 patients (Fig 2). The patients in this study did not have EMG/nerve conduction velocity test after surgery. Although EMG is diagnostically helpful,^11^^,^^12^ the improvement over time on serial EMG examination may^13^ or may not^12^ correlate with clinical outcome.^14^

## DISCUSSION

We^8^^-^10 and other investigators^15^^-^17 have demonstrated that surgical release of LTN and spinal accessory nerve^18^ as a simple and effective treatment that significantly improves winging scapula and shoulder AROM,^8^^-^10 which relieves pain and provides complete motor recovery,^15^ when performed within the first 12 months of the paralysis.^15^ In addition, in our previously published meta-analysis report,^8^ we compared and analyzed the outcomes of long thoracic or spinal accessory nerve decompression and neurolysis with muscle transfer procedures and found that nerve surgical procedures are effective techniques in correcting winging scapula in comparison with muscle and tendon transfer operations.

Vastamäki et al^19^ recently reported how etiology influences long-term outcome of LTN damage. They found that LTN-related palsies caused by infection recover better and those caused by surgery in the body or anesthesia^19^ recover most poorly. These palsies caused by acute trauma or acute or chronic overuse of upper extremities had the same recovery period as other patients. Etiology of LTN injury had less influence on long-term outcomes. We did not have any patient in this study who had LTN injury and winging scapula due to infection. Our patients’ outcome data and recovery also substantiate the findings of Vastamäki et al.^19^ Our data confirm that etiology did not influence significantly in our patient outcomes after surgical LTN decompression treatment.

Mechanism of LTN injury is generally mechanical in origin. Most of our patients in this study had sports-related injuries, and these are mechanical in nature. Glenohumeral instability, scapula winging, proximal humeral physis, and shoulder pain are important to recognize and address early, as these can cause more severe complications later in these growing teen and preteen athletes and other young patients.

## CONCLUSION

Both LTN decompression and neurolysis significantly improved scapular winging in all 16 teen patients in our present study, producing “excellent” shoulder movements in 15 patients (94%) and “good” result in one patient (6%).

## Figures and Tables

**Table 1:** Demographic of teen patients with winging scapula who had long thoracic nerve decompression and neurolysis*

Patient #	Age at surgery, y	Onset to surgery, y	Cause
1	16	0.2	Football
2	17	2.0	Fall/accident
3	17	1.0	Fall/accident
4	15	3.0	Sports-related injury
5	16	1.0	Accident
6	19	1.0	Unknown
7	18	2.0	Fell during soccer
8	19	0.9	Lifting weights
9	19	1.0	Unknown
10	19	0.4	Motor vehicle accident
11	15	0.5	Playing softball
12	17	1.0	Playing baseball
13	17	0.2	Unknown
14	16	2.0	Cheer stunt
15	14	0.9	Cheerleading—tossing
16	18	4.0	Accident

*Three accidents, 2 falls, 2 unknown, 9 sports injury.

**Table 2:** Excellent recovery in shoulder movements after long thoracic nerve decompression and neurolysis in teen patients who had winging scapula*

Patient #	Preoperative flexion, °	Preoperative abduction, °	Extent of SW	Postoperative flexion, °	Postoperative abduction, °	Extent of SW	Follow-up months
1	120	180	3	180	180	4	7
2	90	120	1	180	180	4	18
3	120	180	2	180	180	4	11
4	120	180	1	180	180	4	7
5	120	120	1	180	180	3	20
6	180	120	2	180	180	4	8
7	90	90	1	120	90	3	12
8	180	180	2	180	180	4	56
9	180	180	3	180	180	3	14
10	120	180	2	180	180	4	2
11	120	120	1	180	180	4	5
12	180	180	1	180	180	4	2
13	120	120	1	180	180	3	4
14	120	120	1	180	180	4	3
14	90	90	1	180	180	4	36
16	90	90	1	180	180	3	11
Mean	128	138	1	176	174	4	14
SD	35	37	1	15	23	0	15
*P*				7E-05	2E-03	3E-08	

*Extent of SW range, 1-4: 1, severe; 2, moderate; 3, mild; 4, normal. SW indicates scapular winging.
